# Protein motion in the nucleus: from anomalous diffusion to weak interactions

**DOI:** 10.1042/BST20170310

**Published:** 2018-07-31

**Authors:** Maxime Woringer, Xavier Darzacq

**Affiliations:** 1 Department of Molecular and Cell Biology, Li Ka Shing Center for Biomedical and Health Sciences, CIRM Center of Excellence, University of California, Berkeley, CA 94720, U.S.A.; 2 Unité Imagerie et Modélisation, Institut Pasteur, 25 rue du Docteur Roux, 75015 Paris, France; 3 Sorbonne Universités, CNRS, F-75005 Paris, France

**Keywords:** molecular interactions, nuclear transport, protein motion, transcription factor

## Abstract

Understanding how transcription factors (TFs) regulate mammalian gene expression in space and time is a central topic in biology. To activate a gene, a TF has first to diffuse in the available space of the nucleus until it reaches a target DNA sequence or protein (target site). This eventually results in the recruitment of the whole transcriptional machinery. All these processes take place in the mammalian nucleoplasm, a highly organized and dynamic environment, in which some complexes transiently assemble and break apart, whereas others appear more stable. This diversity of dynamic behaviors arises from the number of biomolecules that make up the nucleoplasm and their pairwise interactions. Indeed, interactions energies that span several orders of magnitude, from covalent bounds to transient and dynamic interactions, can shape nuclear landscapes. Thus, the nuclear environment determines how frequently and how fast a TF contacts its target site, and it indirectly regulates gene expression. How exactly transient interactions are involved in the regulation of TF diffusion is unclear, but are reflected by live cell imaging techniques, including single-particle tracking (SPT). Overall, the macroscopic result of these microscopic interactions is almost always anomalous diffusion, a phenomenon widely studied and modeled. Here, we review the connections between the anomalous diffusion of a TF observed by SPT and the microscopic organization of the nucleus, including recently described topologically associated domains and dynamic phase-separated compartments. We propose that anomalous diffusion found in SPT data result from weak and transient interactions with dynamic nuclear substructures, and that SPT data analysis would benefit from a better description of such structures.

## Introduction

**Figure 1.**
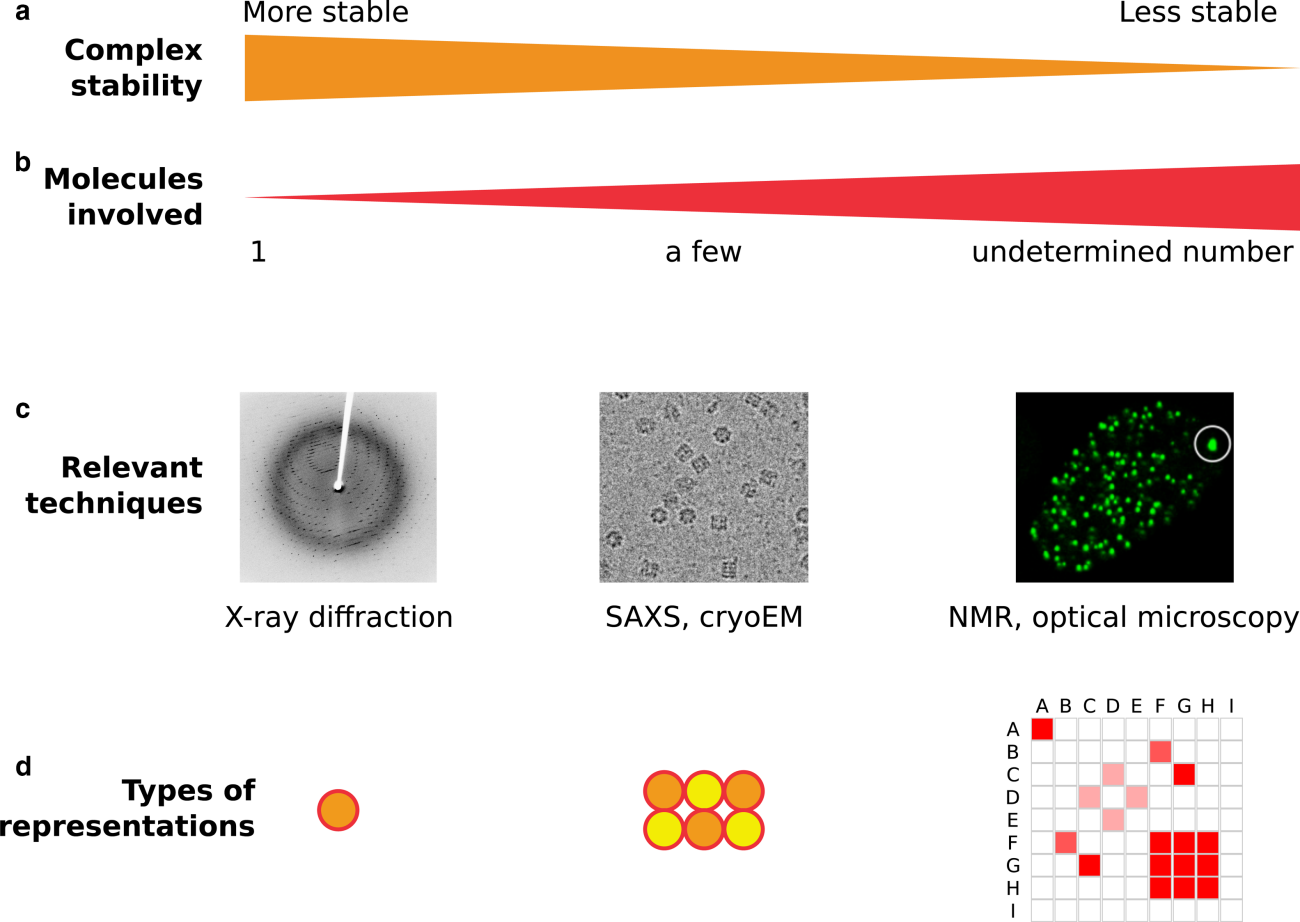
Diversity of weak interactions and example techniques to study them. Biological interactions cover a wide spectrum in terms of complex stability (**a**) and number of molecules involved (**b**). This spectrum spans from stable protein complexes that can be purified and further imaged by techniques such as X-ray diffraction (*left*) to very labile, transient interactions that can involve thousands of proteins in vivo, whereas none of the interactions can be captured by traditional biochemistry (*right*). As one goes from one end of the spectrum to the other end, distinct sets of techniques (**c**) and types of representations (**d**) are needed. For instance, as the valency of interactions increases from a few strongly interacting partners to many weakly interacting partners, new graphical representations are needed, since traditional schematics representing macromolecular complexes whose stoichiometry is known as the juxtaposition of monomers (center) become difficult to read when depicting one protein weakly interacting with dozen of partners (right). In that case, matrices of pairwise interactions between proteins A-I might be more relevant. SAXS: Small-Angle X-Rays Scattering, NMR: Nuclear Magnetic Resonance.

Mammalian gene expression and its regulation take place in the nucleus, a highly complex and subcompartmented organelle. Interactions strengths between nuclear constituents span several orders of magnitude, from covalent bounds to ‘strong’ noncovalent interactions. These interactions lead to the formation of macromolecular structures, either stable (Figure 1, left; for instance double-stranded DNA or biochemically purifiable macromolecular complexes such as the ones involved in gene expression) or transient but specific, leading to preferential associations of classes of proteins (Figure 1, right).

The regulation of transcription is of utmost interest as it is central not only to developmental biology, but also to cancer biology, drug screening, etc. To express a mRNA, a macromolecular complex constituted of several subunits and dozens of proteins, the preinitiation complex, is first to assemble at the promoter of a gene in a time- and space-specific manner [1]. This complex is able to robustly integrate transient signals such as the ones mediated by proteins’ binding to *cis*-regulatory sequences such as enhancers [2].

**Glossary**

**anomalous diffusion** a phenomenon occurring when a molecule explores over time a volume lower than predicted by Brownian diffusion. It is usually characterized by a sublinear growth of the mean squared displacement (MSD) as a function of time (*MSD*(*t*) ∼ *t^α^* with *α* < 1), with *α* the anomalous diffusion exponent.
**compact exploration** universal mode of diffusion in which the exploration of the diffusing molecule is local and distance-dependent and a given site is explored repeatedly over time, in a highly recurrent manner. Within the compact exploration mode, several strengths of compaction can be distinguished.
**facilitated diffusion** biophysical phenomena allowing a molecule to find its target faster than predicted by traditional, 3D free diffusion. This includes diffusion on a surface of reduced dimensionality such as DNA.
**fractal kinetics** type of kinetic reactions happening within a reactor that is not well-stirred. This notably includes most reactions happening on a surface of reduced dimensionality. Fractal kinetics are characterized by a progressive segregation between reagents and products. The kinetic rate *k* of the reaction is time-dependent.
**fractals** structure exhibiting (statistical) self-similarity, that the (statistical properties of the) structure re main similar at various zoom levels. Fractals can be described by their (potentially non-integer) fractal dimension *d_f_* . The fractal dimension of an object describes its space-filling property. Many types of biological objects exhibit fractal properties, such as the branching pattern of the lung, or the hierarchical folding of DNA in the nucleus.
**free diffusion** (also termed Brownian diffusion) chracterizes the motion of a particle in a fluid arising from thermal agitation only. Seen as a “null” model in SPT.
**non-compact exploration** universal mode of diffusion in which the exploration is global, and every site on the structure has a constant probability to be explored (distance independence).
**phase separation** a state of matter in which part of the soluble protein fraction segregates into a liquid or liquid-like droplet.
**surface of reduced dimensionality** an object whose fractal dimension *d_f_* < 3, meaning that it exhibits some properties similar from the ones of 1D or 2D structures.
**weak interactions** (in this review) interactions that are usually too short-lived to be captured by traditional biochemistry techniques, that typically involve one or several wash step, during which proteins interacting specifically but transiently get diluted and washed out.

More mechanistically, the assembly of such a complex can be characterized by a set of chemical reactions describing the progressive recruitment of factors and subunits. A kinetic rate k can be associated to each of these reactions. Traditional biochemistry and *in vitro* experiments have been the methods of choice to investigate such complex processes. Most biochemical techniques involve purification steps allowing to reveal strong, noncovalent interactions such as the ones occurring in a stably assembled complex [3,4] (Figure 1, left). Then, further quantification of stoichiometry and affinity constants became possible, progressively building a network of interacting proteins, usually represented as a graph with nodes linked with arrows.

Within this framework, the understanding of gene expression regulation reduces to elucidating how external factors [including transcription factors (TFs)] affect the kinetic constants k. Although it can be assumed that kinetic rates are characterized only by the nature and concentration of enzyme, substrate, and cofactors, it was shown in 1906 by von Smoluchowski [5] that the kinetic rate of a well-mixed, diffusion-limited reaction can be decomposed as k=4πDa. Thus, the kinetic rate k is a function of both the cross-section of interaction a (reflecting the chemical properties of the partners and usually studied by biochemical approaches) and the diffusion constant D of the species.

Since D is determined by the local environment, this finding is striking in the context of gene expression regulation: now the kinetics of one reaction depend on the whole nuclear structure. More specifically, any factor that affects diffusion in any specific or nonspecific way will ultimately influence reaction rates. Indeed, interactions resulting in facilitated diffusion on a substructure (such as a TF on DNA, [6–8]) or (transient) segregation inside a membrane-less compartment in a phase-separated manner [9,10] can all be seen under the unifying framework of diffusion on a surface of reduced dimensionality. Diffusion on surfaces of reduced dimensionality yields kinetics that are qualitatively different from in free, 3D diffusion and leads to potentially dramatically increased reaction rates [11].

Anomalous diffusion, a phenomenon occurring when a molecule explores a volume lower than predicted by diffusion, affects all proteins inside a cell. Numerous physical models can describe anomalous diffusion [12], and several have been applied to the motion of nuclear proteins. However, many of them only provide a phenomenological description of diffusion, rather than mechanistic insights, and radically distinct models can often fit the available data equally well.

In light of these considerations, it is worthwhile to examine recent discoveries describing either stable subnuclear compartments or their more transient, weak-interactions-induced counterparts to highlight their influence on the diffusion of factors through dimensionality reduction. This includes TADs (topologically associated domains), lamina-associated domains, nucleoli, noncoding RNAs, transcription factories, phase-separated domains, etc. They constitute substructures with a high valency amenable to weak interactions that can qualitatively influence diffusion and target search.

Here, we first review anomalous diffusion models applied to protein motion and relate them to a potential physical generative model. Then, we emphasize recent advances in the characterization of regions of reduced dimensionality in mammalian nuclei, both aspecific through volume exclusion and specific through transient, weak-but-specific interactions. Finally, we propose that these weak interactions shape TF dynamics, and that single-particle tracking (SPT) analysis would greatly benefit from a better understanding of the pairwise interaction map between nuclear proteins.

## Most anomalous diffusion models reflect underlying networks of weak interactions

The technique of choice to investigate protein motion in the nucleus of live cells is light microscopy of fluorescently tagged proteins. Different imaging and modeling modalities have provided significant insights, including fluorescence recovery after photobleaching (FRAP), fluorescence correlation microscopy (FCS) or SPT. In this review, we focus on SPT, because it directly provides access to the dynamics of single diffusing molecules. In a SPT experiment, a small subset of the proteins of interest are imaged and tracked over short times at the resolution of a few tens of nanometers, allowing to resolve isolated single molecules even within clusters of high densities.

In solution, the diffusion coefficient D of a protein is inversely proportional to the hydrodynamic radius of the protein (r) and the viscosity of the medium (*η*) through the Stokes–Einstein relationship $D=k_{B}T/6\pi \eta r$, where $k_{B}T$ reflects thermal agitation, with $k_{B}$ the Boltzmann constant and T the absolute temperature. This description, however, is too simplistic in the complex cellular environment. Indeed, with the exception of inert tracers of small molecular mass [13,14], it is well acknowledged that (a) macromolecules in a cell diffuse much slower than in a medium of comparable viscosity, (b) that complexes of high molecular mass can diffuse faster than small proteins, and (c) that most molecules exhibit anomalous diffusion.

Thus, the diffusion of TFs cannot be described by simple friction/viscosity relationships, and their behavior, perhaps unsurprisingly, has to be seen from the angle of transient interactions with a dense matrix of interactants. In the context of this review, we define transient (or ‘weak’) interactions as interactions that are usually too short-lived to be captured by traditional biochemistry techniques that typically involve one or several wash steps, during which proteins interacting specifically but transiently get diluted and washed out.

**Figure 2.**
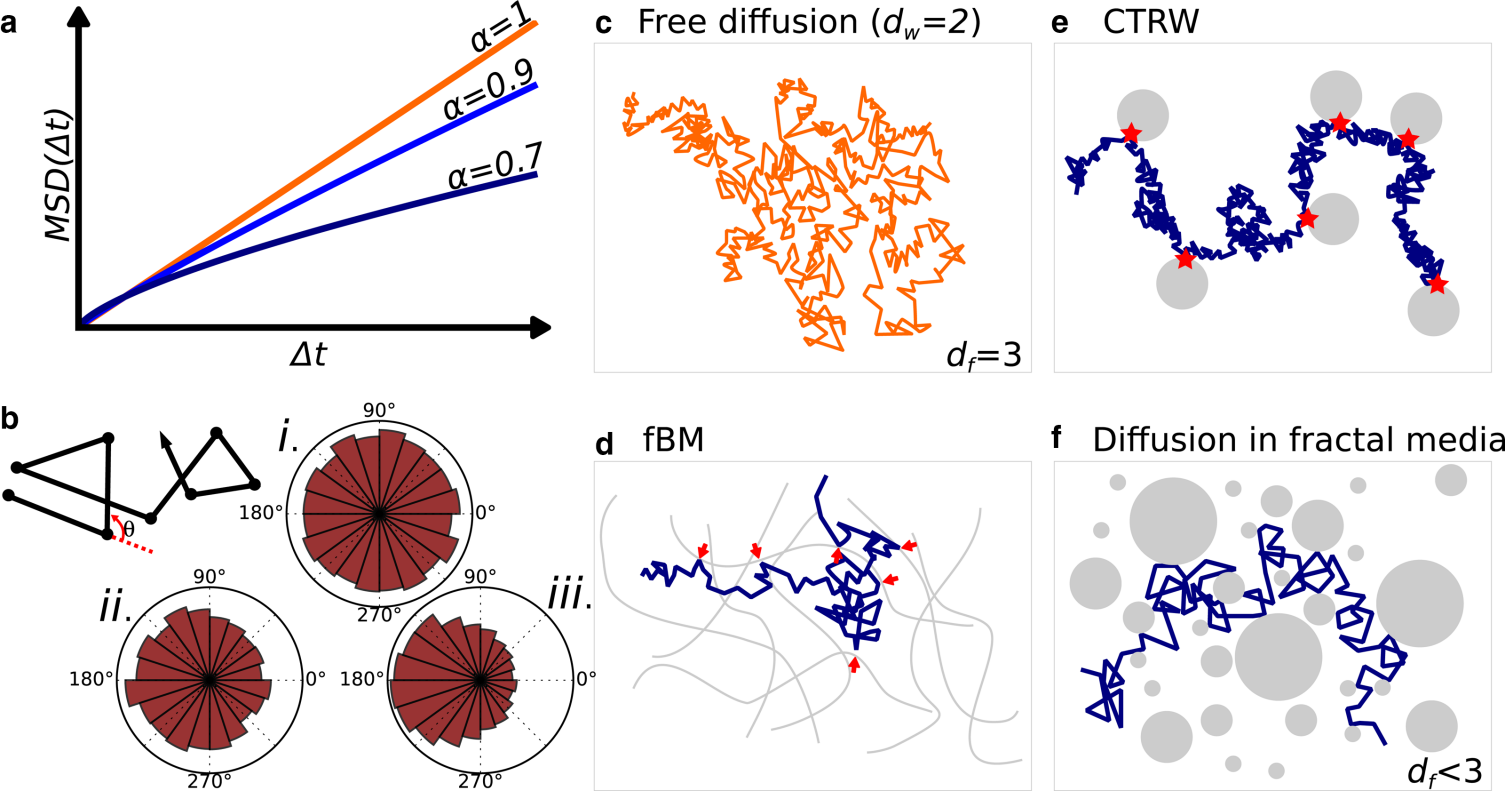
Models of anomalous diffusion and plausible underlying physical structures. (**a** and **b**) Characterizations of anomalous diffusion. (**a**) Sub-linear mean-square displacement plotted as a function of time characterizes subdiffusion, and reflects how a diffusing particle explores space, the degree of anomalous diffusion is characterized by the exponent *α*, the lower the *α* the more subdiffusive the process. (**b**) Free diffusion is characterized by isotropic distribution of angles — subpanel (i) — whereas an anisotropic distribution indicates anomalous diffusion — subpanels (ii) and (iii). (**c**) 3D free diffusion ($d_{w}=2$), as usually encountered in a homogenous media ($d_{f}=3$). (**d**–**f**) Several types of heterogenous media can yield anomalous diffusion, including (**d**) diffusion within a viscoelastic polymer, in which a protein “bounces against” an elastic structure, a process traditionally described by fractional Brownian motion (fBm) and (**e**) free diffusion interspersed by long binding times — red stars, a process called CTRW, and (**f**) diffusion within a fractal media, that is a space obstructed by obstacles of all sizes.

Furthermore, diffusion of many factors does not follow free, Brownian diffusion (Figure 2a,c). Such diffusion is termed anomalous (more specifically, subdiffusive), meaning that the space explored over time by one factor is lower than expected by free diffusion (reviewed in [12,15]). Anomalous diffusion is usually characterized by a sublinear growth of the mean squared displacement (MSD) as a function of time (that is, MSD$(t)∼t^{\alpha }$ with α<1; Figure 2a) and *α* is called the anomalous diffusion exponent. This anomalous diffusion exponent does not fully characterizes the type of diffusion and additional metrics, such as the distribution of translocation angles (Figure 2b), provide valuable information about the dynamics. Anomalous diffusion of proteins has been fitted with success by phenomenological anomalous diffusion models, and include fractional Brownian motion (Figure 2d) [16–19], continuous-time random walks (CTRW; Figure 2e) [20,21] and diffusion in fractal media (Figure 2f) [22–24]. Although useful as phenomenological descriptions, these models are often agnostic regarding the underlying reality of the process. In any case, the explanation of diffusion has to rely on physics and chemistry of the nucleus.

From a physical perspective, proteins can adsorb and diffuse on nuclear substructures. When this happens, the exploration properties of the protein are universally given by two parameters: first, the dimension of the random walk $d_{w}$ (deduced from the anomalous diffusion exponent: $d_{w}=2/\alpha$, i.e. the scaling of the MSD: $\mathrm{MSD}(t)∼t^{2/d_{w}}$), and second, the dimension of the space available to diffuse $d_{f}$. $d_{f}$ can be integer ($d_{f}=1$ for instance for sliding on DNA without jumps, $d_{f}=2$ for a factor freely diffusing on the surface of a subcompartment), or noninteger, a feature that characterizes self-similar structures, that is, fractals (Figure 2f). For instance, highly obstructed media or dense environments where the available volume is reduced to small pores are often accurately described by their noninteger fractal dimension $d_{f}$. Such a case can occur in dense subcompartments such as phase-separated domains. For the sake of this review, we will denote structures of $d_{f}<3$ as structures of *reduced dimensionality*.

Depending on $d_{f}$ and $d_{w}$, the motion of the protein falls into two universal categories, termed compact and noncompact [25–27]. In a compact exploration ($d_{w}>d_{f}$), exploration is local and distance-dependent and a given site is explored repeatedly over time, in a highly recurrent manner. Conversely, in a noncompact exploration ($d_{w}<d_{f}$), the exploration is global, and every site on the structure has a constant probability to be explored (distance independence); the exploration is nonrecurrent (transient). For instance, a particle freely diffusing has a $d_{f}$ of 2. When diffusion takes place in a 3D space ($d_{f}=3$) the particle tends not to revisit sites, adopting a noncompact exploration (indeed, $d_{w}<d_{f}$). Conversely, a particle in free, Brownian diffusion ($d_{w}=2$) constrained to diffuse in 1D ($d_{f}=1$; hypothetically along a DNA fiber) will repeatedly sample the same sites (compact exploration, $d_{w}>d_{f}$). Consequently, target search times are decreased and reaction rates are increased in the compact case.

Structures of reduced dimensionality, including fractals, emerge naturally from various processes, including diffusion-limited aggregation and hierarchical assembly of macromolecular scaffolds, such as the multi-scale organization of chromatin. The goal of the next sections is to highlight a few structures of reduced dimensionality in the nucleus and how they influence kinetics of TFs.

## Steric hindrance in the nucleus

Far from constituting a homogeneous medium, the nucleus is a highly organized and subcompartmentalized organelle. The main organizing structure, chromatin, constitutes ∼10–30% of the nuclear volume [28,29] and probably accounts for a significant part of the diffusion slowdown [30]. Since every molecule has to slalom around a dense and heterogeneous chromatin environment, diffusion is impaired. Note that, however, similar diffusion coefficients are usually observed in the cytoplasm and the nucleoplasm [31], suggesting that protein crowding can also account for diffusion slowdown ([32,33] and [23] for a discussion).

Over the past years, organizing principles of chromatin have emerged: at large scale, the genome is segregated in chromosome territories and regions of heterochromatin/euchromatin, lamina-associated domains at the periphery and nucleoli lying more at the center. At higher magnification, chromatin is organized in areas of preferential interactions such as A/B compartments and TADs that reflect the functional organization of chromatin [34].

Overall, although highly heterogeneous, chromatin in the mammalian nucleus is well described by a self-similar, fractal structure that occupies a nonzero volume. This was initially postulated [35], and later evidenced by spectroscopic [36,37], genomic [38,39] and imaging techniques [18,23,40–42].

As a consequence, factors diffusing in the available volume are constrained by this structure [43], possibly experiencing diffusion in a medium of reduced dimensionality, as evidenced by numerous reports [23,44]. In a model where only volume exclusion happens, proteins of the same size and shape should have the same diffusion coefficient. Thus, the embedding structure of the nucleus only sets a lower bound on the level of anomalous diffusion that can be observed.

Several lines of argument point to the fact that although steric hindrance induces a significant decrease in the diffusion coefficient (D), its effect on anomalous diffusion (*α*) is mild for proteins of weight <150 kDa (that is, about the weight of a histone octamer wrapped with 150 bp DNA). Indeed, FRAP experiments performed with protein or nonprotein tracers of increasing molecular mass suggest that low molecular mass tracers diffuse almost freely in the nucleus, allowing to infer a viscosity close to the one of water [13]. At molecular mass >150 kDa, anomalous diffusion becomes more prominent [14,23,31], sometimes leading to particles occasionally being trapped in the chromatin mesh. This effect is consistent with the relatively limited volume occupied by chromatin [28,29] that can trap big complexes but not smaller proteins.

In conclusion, although volume exclusion by chromatin and other nuclear constituents is real, it affects all proteins of the same size in a similar manner. In contrast, a protein weakly but specifically interacting with such a structure (for instance, TFs sliding/hopping on DNA [6,45–48]) will immediately show a much higher level of anomalous diffusion. Furthermore, even without considering a fractal structure, simple dimensionality reduction to 1D or 2D can yield nontraditional kinetics termed fractal kinetics [11,49,50]. Fractal kinetics are characterized by a time-dependent reaction rate: for a A+A→products reaction, the reaction rate $k_{\mathrm{app}}$ can be expressed as $k_{\mathrm{app}}=kt^{-h}$, with h that depends on the (fractal) dimension of the space. Fractal kinetics arise when the reaction cannot be assumed to be well stirred, which is the case of diffusion on surfaces of reduced dimensionality. For instance, fractal kinetics in 2D could occur by weak interaction with the nuclear lamina, Figure 2b. All in all, weak and transient interactions shape the nuclear landscape and can give rise to emergent structures and properties, as exemplified in the next section.

## Weak interactions in the nucleus

Unlike inert tracers whose diffusion is only determined by volume exclusion, proteins have both a relevant shape and charge pattern that determine their interaction landscape and thus their diffusive properties. These noncovalent interactions are obviously crucial to form biochemically stable complexes such as the transcription preinitiation complex or the spliceosome (Figure 1, left), but also to form dynamic emergent structures of reduced dimensionality upon which TFs can transiently adsorb and diffuse (Figure 1, right). Under this model, proteins do not form stable complexes anymore, but rather have a high number of weakly interacting partners. Indeed, simulation studies have shown that phase separation yielding structures of reduced dimensionality can occur under very minimal hypotheses, such as weak overall protein-protein attraction [51,52]. In this context, the traditional representation of protein-protein interaction networks as graphs and arrows becomes less informative as the protein network gets fully connected, and can be replaced by representations such as pairwise interaction matrices (Figure 1d, right) [53]. Furthermore, the list of proteins exhibiting phase separation (that is, a state of matter in which part of the soluble protein fraction segregates into a liquid or liquid-like droplet) *in vitro* or *in vivo* is quickly growing, supporting the vision that the emergence of structures of reduced dimensionality is closer to a general organizing principle than an anecdotal biophysical phenomenon, and some of them were linked with transcriptional regulation [9,54–57].

First, transient structures of reduced dimensionality, including protein aggregates, phase separated domains or subnuclear compartments require at least one *multivalent partner* that can nucleate the aggregation. As such, many abundant constituents of the mammalian nucleus have been shown to nucleate a structure of reduced dimensionality. These structures include the formation of nucleoli in which diffusion is highly constrained, phase-separation of heterochromatin protein 1 domains (HP1; [9,10]) and highly active chromatin domains, whose center has reduced accessibility to diffusing proteins, restricting most diffusing proteins to the surface of the domain [57]. The constituents include low complexity protein domains that constitute the majority of the mammalian proteome [58,59], especially TFs [60], repeated DNA [61] or RNA sequences [62–64] or small amphiphilic molecules [65,66]. Once nucleated, these structures can have a highly tortuous, potentially fractal architecture, and can serve as a scaffold that can weakly trap other proteins, leading to anomalous diffusion.

Second, the partners have to exhibit compatible interactions: it is chemically unlikely that both highly charged and hydrophobic proteins will coexist in the same structure without the help of additional compounds acting as counterions [67], setting the basis of a ‘grammar of interactions’ [68], that is being progressively deciphered [56,66,69–72].

Third, structures of reduced dimensionality emerging from weak interactions exhibit the following properties: (1) they usually exist as an extremely dynamic equilibrium rather than a stable structure [10,64,73], and can thus be at the same time prevalent in the nucleus and hard to purify by traditional biochemistry that preferentially capture stable interactions. (2) Moreover, they emerge from a dynamic mesh of pairwise chemical interactions. They can show a high level of specificity, and several structures of reduced dimensionality can coexist in the same nucleus without intermixing [55,57,61,67,74]. Furthermore, the number and spatial relationships of such structures is only limited by the combinatorics of chemical interactions. (3) Finally, these structures can be regulated by the well-studied post-translational machinery of eukaryotic cells. For instance, phosphorylation of one of the proteins involved in such structure can trigger the timely disassembly of the whole structure and free all the factors interacting with it [9,55,75–77]. All those factors will then exhibit a dramatically different dynamics and target search properties, potentially switching from a compact exploration mode to a noncompact one. As such, a specific (and potentially functional) group of factors can be regulated at once by modulation of the post-translational modifications of one scaffolding protein [61,63].

The characterization of structures of reduced dimensionality emerging from weak interactions is still in its infancy, but appears more and more strongly as a clear organizing principle of mammalian nuclei. These structures create the matrix upon which fast-diffusing factors can specifically and transiently bind, diffuse and unbind, thus dynamically shaping the ‘diffusion landscape’ of the whole transcriptional machinery.

Even though live imaging approaches specifically characterize the behavior of one single factor, they are blind to all these substructures. Indeed, SPT reflects the dynamics of proteins transiently interacting with those structures of reduced dimensionality and one TF potentially visits several of them in the span of a few tens of milliseconds [78]. Such complex behavior therefore appears macroscopically as various kinds of anomalous diffusion.

## Perspectives: seeing beyond the dots

In a complex mammalian nucleus, the diffusion of a TF is ruled by transient interactions with underlying structures of reduced dimensionality. From a more general perspective, the question arises of how gene expression regulation processes relate to the multiplicity of structures of reduced dimensionality?

**Figure 3.**
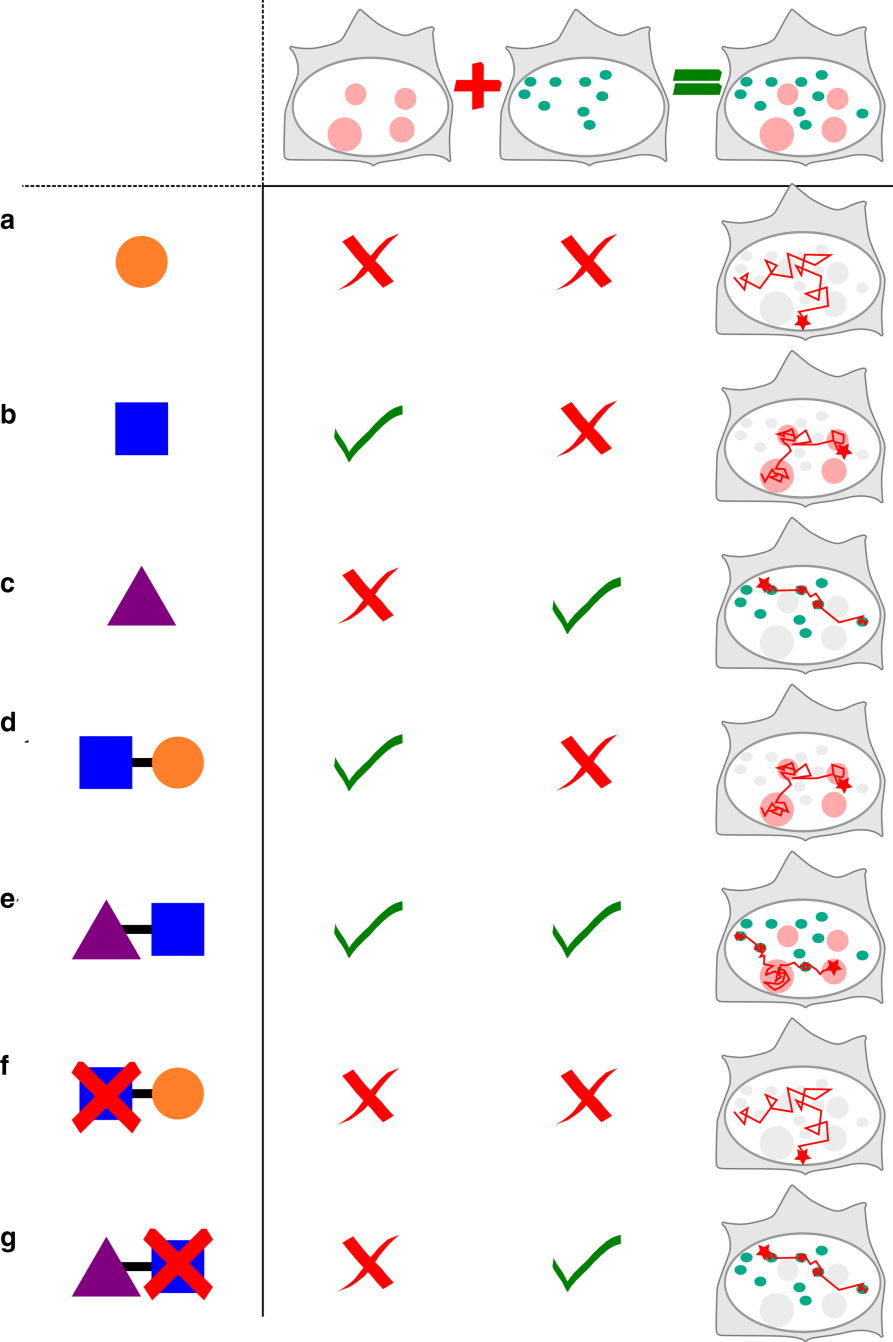
Weak interactions of individual protein domains shape TF dynamics. (**a**)–(**c**) Individual protein domains have specific transient interactions: the round domain (**a**) does not interact with any particular structure (represented by the two columns of the table), (**b**) the square domain interacts with a given pink structure (first column) and (**c**) the triangle domain interacts with the green structures (second column). This results in domain-specific dynamics (third column). (**d** and **e**) When protein domains are combined within a protein or TF, the observed SPT is a mixture between the interactions of each single domain. (**f** and **g**) When individual domains are mutated, the protein loses some transient specific interactions, and its dynamics can dramatically change (compare panels **e** and **g**).

Proteins often harbor several domains (Figure 3), holding the potential to interact successively and repeatedly with multiple classes of structures of reduced dimensionality. Thus, depending on its interaction domains, a TF will ‘see’ a different landscape and will interact with some structures, whereas other factors will either be excluded or cross them without any additional interactions than limited steric hindrance. In this respect, the nucleus can be described as a ‘multiverse’, in which some factors coexist in the same physical space but exhibit radically distinct dynamics and interactions (Figure 3).

Furthermore, structures of reduced dimensionality have been proved to be functionally relevant. For example, the dynamic and regulated switching of a TF between structures of reduced dimensionality determines its dynamics (Figure 3a–e) and function. It has been shown that TF exhibit radically different dynamics before/after a post-translational modification [79,80], or an artificial deletion of a domain (Figure 3f–g), [8,81–85]. In that case, the observed diffusion will be arising from the remaining interactions from the other interaction domains, or ultimately from simple volume exclusion [86].

Although theoretical and experimental support for the importance of weak interactions as an architectural principle of the nucleus and gene expression regulation is being actively investigated, several questions remain unaddressed:

First, how many distinct types of structures of reduced dimensionality exist? Since the numbers of types of low-complexity domains are likely to be limited, one can expect that a limited number of such structures actually coexist at a given time in a nucleus [71]. This implies that the SPT dynamics of TFs will fall in a limited number of categories, which in turn is determined by their combinatorial interactions with one or several of these structures. To take into consideration such processes paves the way for a higher-order understanding of gene expression regulation and key transitions occurring, for instance, during mitosis or development.

Second, can we determine the pairwise interaction matrix between low-complexity protein domains, which would allow us to derive predictive dynamics of a given TF modification? Ideally, such matrix will encompass all known low-complexity domains, but also abundant multivalent RNAs and DNA sequences, and each element of this matrix will reflect the affinity between two domains under physiological conditions (Figure 1d).

Third, how much detail is required to describe these structures of reduced dimensionality? Is the pairwise interaction between protein domains a good approximation of the properties of the nucleus? Conversely, one can imagine substructures of reduced dimensionality arising from interactions more complex than simple pairwise-interactions. Indeed, it is widely known that cooperativity plays a role in the assembly of many more or less stable macromolecular structures [87], including some phase-separated domains [67].

Fourth, how do key biological transitions such as differentiation intertwine with these structures of reduced dimensionality? In a similar way, as pluripotency or cell-type specific TF networks have been identified, can pluripotency or cell-type specific structures of reduced dimensionality be evidenced, integrating the expression levels of TFs and providing a framework to better understand such key processes?

To answer those questions, our understanding of nuclear processes need to be drastically expanded. Hitherto, a dynamic picture of spatially segregated factors, together with their interaction matrix, is currently missing. Promising tools to access those parameters include quantitative FRET [88], in cell NMR [89–91], low-photons SPT [92], tracking FCS [93], spatially resolved FCS [94] and computational methods [72,95].

## Conclusion

Although the so far identified key players in gene expression regulation are biochemically stable complexes that can be purified using traditional methods, increasing evidence suggest that higher-order, weaker-interaction structures, acting as structures of reduced dimensionality, play a central role in transcriptional regulation. They do so by providing a remarkably versatile way of specifically and timely regulating TF target search dynamics and thus gene expression. All in all, it appears that the functional properties of the nucleus are shaped by not only stable macromolecular complexes but also transient structures (arising from a continuum of weak interactions that might seem spurious). In this context, the saying from Heraclitus makes probably more sense than ever: ‘The fairest order in the world is alike a heap of random sweepings’.

## Abbreviations

CTRW: continuous-time random walks
FCS: fluorescence correlation microscopy
FRAP: fluorescence recovery after photobleaching
MSD: mean squared displacement
SPT: single-particle tracking
TADs: topologically associated domains
TF: transcription factor
